# Interactions of Comorbidity and Five Simple Environmental Unhealthy Habits Concerning Physical and Mental Quality of Life in the Clinical Setting

**DOI:** 10.3390/ijerph18189590

**Published:** 2021-09-12

**Authors:** Diego Martínez-Urbistondo, Rafael Suarez del Villar, Omar Ramos-Lopez, María Agud Fernández, Ramón Costa Segovia, Andrea Domínguez, Rocío García de la Garza, María López-Cano Gómez, Laura Prósper Ramos, Rodrigo San-Cristobal, Lidia Daimiel, Paula Villares Fernández, Jose Alfredo Martinez

**Affiliations:** 1 Internal Medicine Department, Hospital HM Sanchinarro, HM Hospitales, 28050 Madrid, Spain; rafasdvc@gmail.com (R.S.d.V.); mariaagud@gmail.com (M.A.F.); ramoncostasegovia@gmail.com (R.C.S.); adominguez@gmail.com (A.D.); rochire@gmail.com (R.G.d.l.G.); marialopezcano.gomez@gmail.com (M.L.-C.G.); la_prosper@hotmail.com (L.P.R.); pvillares@hmhospitales.com (P.V.F.); 2 Medicine and Psychology School, Autonomous University of Baja California, Tijuana 22390, Mexico; os_mar6@hotmail.com; 3 Precision Nutrition and Cardiometabolic Health Program, IMDEA Food Institute, CEI UAM + CSIC, 28049 Madrid, Spain; rodrigo.sancristobal@imdea.org (R.S.-C.); jalfredo.martinez@imdea.org (J.A.M.); 4 Nutritional Control of the Epigenome Group, Precision Nutrition and Obesity Program, IMDEA Food Institute, CEI UAM + CSIC, 28049 Madrid, Spain; lidia.daimiel@imdea.org; 5 CIBERobn, Carlos III Institute, 28029 Madrid, Spain

**Keywords:** health-related quality of life, comorbidity, unhealthy habits, lifestyle, physical QoL, emotional QoL

## Abstract

The objective of this study was to examine the interactions between comorbidity and five lifestyle single habits concerning different subscales of quality of life (QoL). For the study, 302 patients were consecutively recruited at the internal medicine department of a tertiary teaching hospital. Lifestyle habits, comorbidities and QoL were recorded according to validated questionnaires. Five single unhealthy habits, such as tobacco consumption, dietary intake of ultra-processed pastries, raw nuts or carbonated drinks, sleep time and physical activity patterns were selected according to previously published data. The main outcomes of the study were the scores of the eight subscales of the SF-36 QoL survey. The aggregate of unhealthy habits showed statistically significant association to every category in the SF-36 questionnaire, both in the univariate and the multivariate analysis when adjusting by age, sex and comorbidity. An interaction was found between comorbidity and unhealthy habits in both physical and mental summaries of SF-36. In conclusion, the lifestyle assessment according to five unhealthy habits is associated with a worse QoL. The interaction between comorbidity and unhealthy habits is especially clear in diseased patients due to the interplay between illness and lifestyle in the prediction of QoL.

## 1. Introduction

Quality of life (QoL) is defined as “the perception of the position in life of individuals in the context of the culture and value systems in which they live and in relation to their goals, expectations, standards and concerns” [1]. In this context, QoL is the synthesis of the interplay between different health aspects such as disease, lifestyle, sleep, nutrition and genetics in the perception of own wellbeing [2,3,4]. QoL traditionally encompasses a comprehensive concept influenced by complex behaviors accompanying the persons’ physical health, psychological state and level of independence, including social relationships to salient environmental features, whose interactions with disease has been rarely monitored with a holistic scope [5].

On the other hand, there is no unanimous definition of wellbeing, but there is general agreement that the well-being principle involves the occurrence of constructive reactions and feelings, the lack of negative manners, enjoyment with life attainments, and specifically plenty of energy attitudes and overall metabolic health [6]. Finally, health-related quality of life (HRQoL) is a suitable estimator of overall health, quantifying traits concerning the physical and mental health status of individuals, and on the impact of health status on QoL. Thus, HRQoL is usually screened through diverse items of self-perceived health status and physical and emotional measures by providing a comprehensive appraisal of the global problem of preventable diseases, injuries, and disabilities. In fact, a systematic review and meta-analysis demonstrated that a better QoL/HRQoL was related to lower mortality risk, which suggests the utility of these tools in predicting mortality risk in general clinical practice [7]. The quantification of HRQoL can be achieved by the application of different questionnaires [8,9]. In this context, SF-36 has been demonstrated to be an effective test in both research and clinical settings to measure physical and mental HRQoL [10,11,12,13,14,15]. 

Undeniably, there is a direct relationship between disease and HRQoL [16,17,18]. However, the assessment of comorbidity could be obscured by the heterogeneity of illness and the accumulation of different diseases in the same patient. In this scenario, different indexes have been developed to predict survival depending on the individual disease burden. Among these, the Charlson Comorbidity Index (CCI) is widely validated and applied in the acute and the chronic settings [19].

Finally, the interaction between lifestyle and QoL is another important feature for evaluating wellbeing. In this context, multiple lifestyle measures have been evaluated in prospective cohorts, concerning dietary patterns [20], physical activity, measured in different manners [21], sleep duration and quality [22]. Nevertheless, the lifestyle assessment is usually taken into account in healthy subjects, in the impact in the development of disease or in specific diseases. Thus, the day-to-day evaluation of lifestyle and QoL in the real-life medical setting is in an embryonic stage. Some facts that could be related to this phenomenon would be the failure to implement lifestyle measures by general practice counselling, the prioritization of other medical aspects of the patient, the scarce real-life data of lifestyle associated with health improvement and the low cost-effectiveness of the concise assessment of individual lifestyle patterns [23,24]. For these reasons, in order to demonstrate the association of simple habits to QoL as well as the interaction between simple lifestyle measures with disease burden could be of interest in the implementation of precision medicine and for patient health empowerment at any time [25]. 

The objective of this study was to evaluate the impact of lifestyle as represented by a proxy of five common unhealthy habits in the different domains and summaries of SF-36 QoL depending on the morbidity burden of patients in a real-life clinical scenario.

## 2. Materials and Methods

A cross-sectional cohort study was developed including 302 consecutive incoming patients, who attended a medical ambulatory examination in a Spanish tertiary hospital between October 2018 and March 2019 by a group of Internal Medicine specialists to receive medical counselling or prescriptions as well as to be monitored about different morbidities and accepted to fill a validated wellbeing questionnaire including lifestyle variables from different validated surveys, such as Global Physical Activity Questionnaire (GPAQ) and Mediterranean Diet Adherence Questionnaire 14 (MeDiet-14) [10] and SF-36 v2 form [6]. The attending physician used the CCI to collect morbidities [19]. Depression was recorded according to the report of a current diagnosis of depressive disorders by the patient. Of this cohort, 40 out of 302 individuals (13.28%) were excluded due to incomplete filling out of the questionnaire or lack of anthropometric or clinical information.

All the data recorded from patients were used in a clinical setting and for clinical purposes, which add special care of data treatment. This population was recruited as a pilot part of a longitudinal study (ESCAVIDA). The ethics board of our center approved the use of cross-sectional and retrospective collection of data to proof the concepts previously to the development of the prospective part of the study. All patients received an information sheet prior to the recruitment informing about the use of data in research, had contact with a research doctor after completing the questionnaire and did not reject the participation. The study was approved by the center bioethics committee (ESCAVIDA/04). 

An operational assessment was devised to analyze the interactions of common unhealthy habits such as smoking, food and drinks consumption and physical activity traits as well as comorbid conditions on QoL in order to evaluate the interaction between lifestyle and comorbidity in wellbeing perceptions. The objective of the analysis was to predict SF-36 subscales which are Physical QoL, Physical role, Emotional QoL, Mental QoL, Social QoL, Vitality, Pain and General QoL, and SF-36 physical and mental summaries as the main outcome of lifestyle and morbidity. After data recollection for each SF-36 domains, ponderal adjustment was attributed to each subscale to turn the 8 SF-36 categories into the Physical and the Mental SF-36 summaries as previously published [25]. Patients were considered comorbid when they accumulated at least two of the comorbidities in the CCI index. An unhealthy lifestyle pattern was considered as an aggregate accounting the five unhealthy habits: smoking, absence of consumption of raw nuts, unhealthy dietary habits considered as the consumption of ultra-processed products such as pastries or carbonated beverages according to the Mediterranean dietary pattern adherence questionnaire, absence of reported specific weekly time devoted to physical activity and sleeping time less than 7 h. These habits were selected according to previous evidence showing the association of these factors with the development of chronic diseases, mainly in the European population. Moreover, these items are part of the validated Mediterranean dietary pattern adherence questionnaire as well as the GPAQ [7,26]. The association between SF-36 subscales and summaries and habits was assessed in both univariate and multivariate analysis, adjusted by age, sex, physical and mental comorbidities. Then, the interaction between habits and morbidity in the prediction of QoL was assessed.

Conventional statistical tests, including chi-square and T-Student were applied as appropriate. Factorial 2 x 2 ANOVA and regression analysis were performed to evaluate interactions comorbidity, lifestyle and QoL. Multivariate regression models were run considering different SF-36 subscales, using each category as a dependent variable, while confounding variables such as comorbidity and depression and adjusting variables, such as age or sex, were also fitted in the model. To avoid co-linearity, age was included separately from CCI in the multivariate models. Results were considered statistically significant with a *p* value < 0.05. The IBM SPSS statistical package v20.0 (Chicago, IL, USA, 2011) was used to perform the analysis, where the application manual was followed when proceeded.

## 3. Results

The study population had a mean age of 55 ± 17 years with a female participant rate of 52%. A total of 32 patients (12.20%) declared to be current smokers. Unhealthy dietary habits as assigned by ultra-processed pastries or carbonated beverages were found in 109 patients (41.60%), while no raw nuts were consumed by 146 (55.70%). Finally, 89 patients did not declare any physical activity, while a total of 204 patients slept for 7 h or less. The aggregate of unhealthy habits as well as the disease burden of the cohort was distributed as shown below (Table 1). A total of 30 patients reported depression (11.50%). CCI mean of the cohort was 0.91 ± 1.39. Mean and standard deviation of the eight subscales of SF-36 are reported (Table 1). The results for Physical and Mental SF-36 summaries were 65.31 ± 22.44 and 71.91 ± 22.46, respectively.

**Figure 1:**
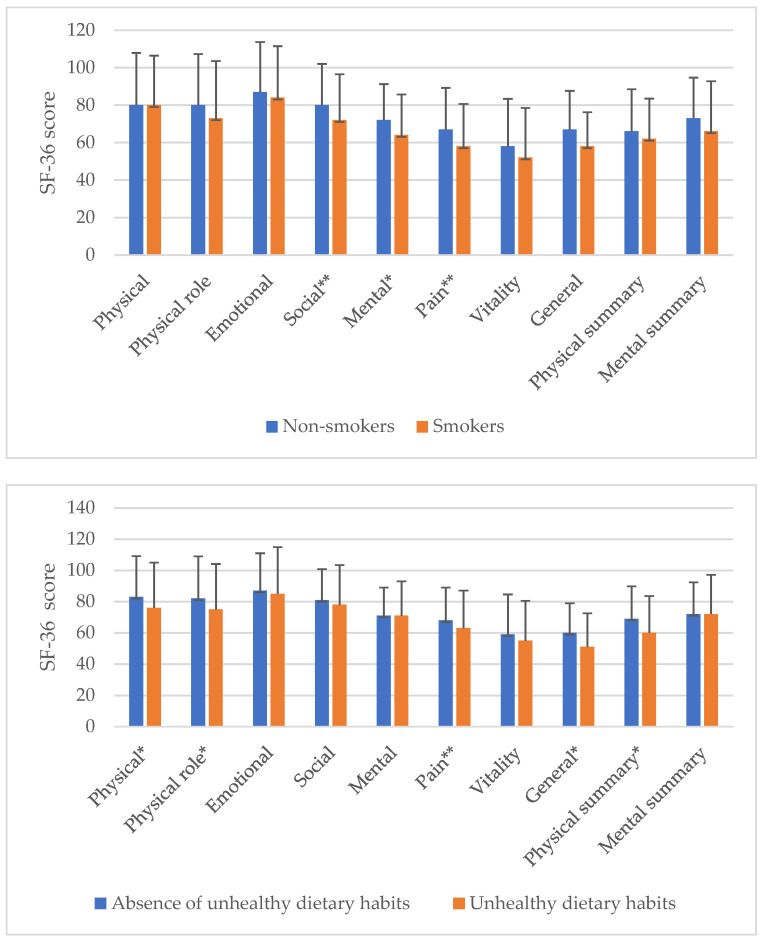

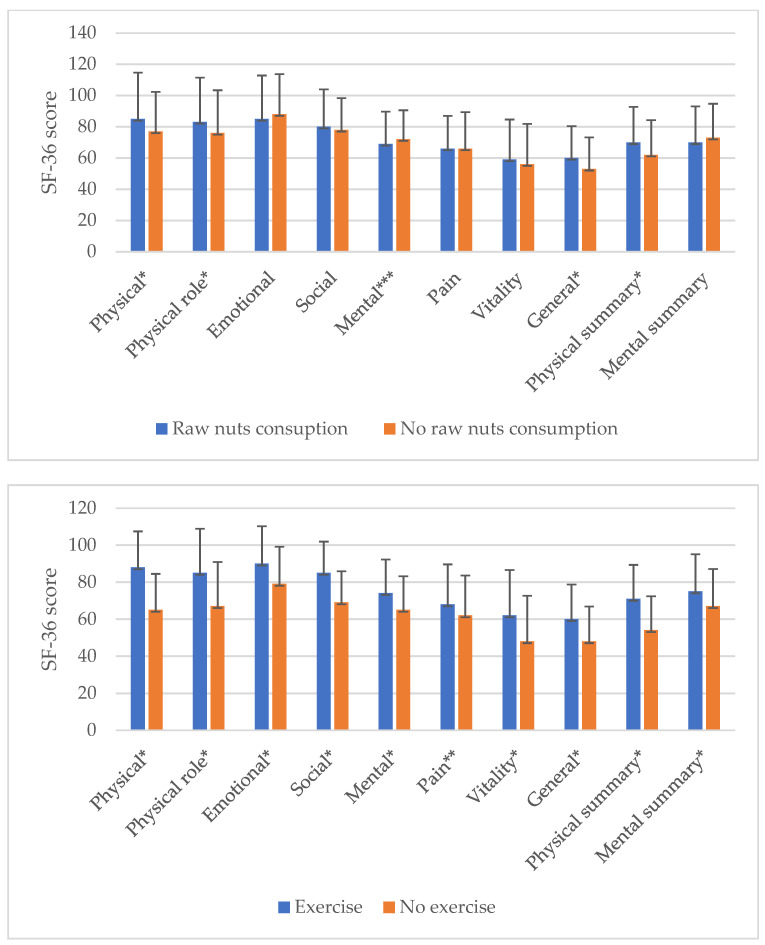
Univariate association of unhealthy habits and QoL SF-36 subscales. * for *p* < 0.05 ** for *p* < 0.10 *** No significant inverse association.

A univariate analysis was performed to assess the association between unhealthy habits and QoL. In the single analysis of each habit, smokers had a worse QoL than non-smokers in Mental QoL (*p* < 0.05), with marginal differences in Social QoL and Pain domains (*p* < 0.10). Unhealthy dietary habits such as frequent consumption of carbonated drinks and ultra-processed pastries had a negative impact on Physical QoL, Physical role, General QoL and Physical component (*p* < 0.05), with a statistical trend in the Pain category (*p* < 0.10). Additionally, the lack of extra fiber in the diet of patients, assessed by the absence of raw nuts consumption, negatively influenced the result in the subscales of SF-36: Physical QoL, Physical role, General QoL and Physical SF-36 summaries (*p* < 0.05). The absence of declared physical activity was associated with a worse QoL in all SF-36 subscales (*p* < 0.05) except for the Pain category (*p* < 0.10). A sleep time below 7 h was related to a worse Mental QoL (*p* < 0.05) and a trend to a lower Mental component score (*p* < 0.10). These results and the statistical analysis are illustrated (Figure 1).

The univariate analysis of aggregated unhealthy habits was also performed (Table 2).

Then, multivariate analysis was performed, adjusting every QoL category by age, sex, CCI score, depression diagnosis, and the aggregate of unhealthy habits. The results of the analysis are shown in Table 3.

**Figure 2:**
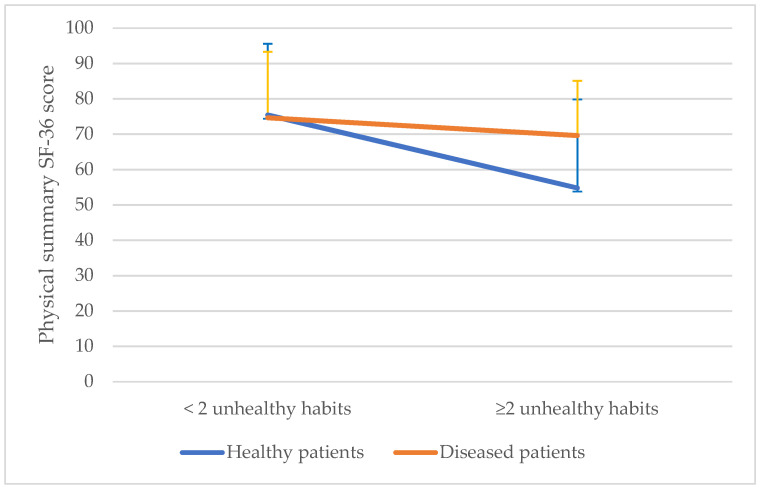
Interaction between morbidity and unhealthy habits in the prediction of SF-36 physical and mental summaries, adjusted by age and sex.

Finally, to assess the interaction between disease and unhealthy habits in the prediction of Physical and Mental summaries of SF-36, two models were fitted. In the first model, an interaction was found in the Physical SF-36 aggregated domain between ≥2 unhealthy habits and the presence of disease according to CCI (*p* for interaction < 0.001). In the second model, depression and ≥3 unhealthy habits were found to interact in the prediction of Mental SF-36 aggregated domain (*p* for interaction 0.016), as shown (Figure 2). 

Adjusted by age and sex. *p* for depression and unhealthy habits < 0.05. *p* for interaction = 0.016.

## 4. Discussion

The HRQoL is emerging as a relevant medical issue, reflecting the overall subjective wellbeing of the patients [18,27]. Indeed, an improvement in the QoL of patients is one objective of current personalized medicine [28]. Nevertheless, the clinical approach to lifestyle must be easy to assess in the clinical setting due to lack of time. In fact, in the current clinical scenario, it is still difficult to introduce lifestyle evaluation in addition to clinical evaluation of present co-morbidities and other chronic disease. In this context, a rapid proxy to life habits using five objective factors with direct impact on health-related quality of life could help the introduction of lifestyle appraisal in the medical day-to-day practice. 

Additionally, wellbeing assessment needs to be adapted to the population heterogeneity, with comorbidity as a keystone in the medical field [17]. Lifestyle adequation to some standards is known to be related to a higher health-related quality of life [4,20,21,22] and the disease burden is associated with a decrease in SF-36 results [17,18,19]. Nevertheless, the impact of the interaction between co-morbidity and lifestyle in HRQoL remains unclear. The results from the present study emphasize the different SF-36 mental and physical composite association to lifestyle depending on morbidity. In fact, morbid patients might be more reactive to lifestyle adherence than apparently healthy individuals in terms of quality of life. These findings might partially explain the low adherence of the general population to habit counselling and could be used in the future as a proof of concept for quantitative objective assessment and interpretation of the lifestyle effect on quality of life measurements. Furthermore, the actual results might enhance precision medicine possibilities and help the promotion of the health empowerment of patients.

Some methodological aspects of the study may reinforce these conclusions. The consecutive recruitment of the population is one of the strengths of the study, with a high adherence to the questionnaire (>85%). The fact that the patients were enrolled during a medical visit is another interesting point of our research, because lifestyle recommendations are usually disseminated through Public Health channels [29] while the development of QoL assessment in clinical practice needs to be supported by real life data [30]. The record of comorbidity by clinicians should also reinforce the quality of disease burden assessment. The use of CCI and a validated lifestyle questionnaire, as well as SF-36 index supports the validity and reproducibility of the study [21,31,32]. The selection of simple yes/no habits in the assessment could facilitate the easy and reliable inclusion of lifestyle anamnesis in the general medical attention to patients.

About the plausibility of the present investigation, lifestyle, comorbidity and QoL interactions have been widely studied [32,33]. Comorbidity was in the core of QoL subscales development [5,10]. Additionally, the five unhealthy habits that were selected in the analysis of the present cohort have all demonstrated their impact in health. Tobacco has been associated with a loss of QoL in different studies, with a dose-dependent effect and an association to depressive states [34]. Dietary habits have an impact on wellbeing, which can be beneficial, as in the case of raw nuts, which have been demonstrated a singular positive effect on preventing cardiovascular disease due to the fat quality and bioactive compounds. In other cases, dietary patterns might be hazardous, such as the consumption of ultra-processed dietary products, which are associated with a higher mortality ratio by all causes or the substitution of water by carbonated drinks, which is directly related to weight gain [35,36,37]. The performance of physical activity in a stable basis and an adequate sleep time have also demonstrated an impact on health in different studies, especially in the elderly, with an effect on energy balance and lipid metabolism [21,38]. The analyses were specifically focused on dietary and physical activity in a morbid measured with validated subscales, such as the Mediterranean dietary pattern adherence questionnaire and the GPAQ, which were retrieved to characterize interactions between QoL with nutrition and physical activity in a rarely apprised setting.

Interestingly, our research shows different impact of habits over QoL subscales. In this context, habits related to dietary pattern are more related to Physical SF-36 features, especially in physical QoL, physical role and general QoL, while mental aspects of SF-36 were more associated with tobacco consumption and sleep [39]. Physical activity was both associated with physical and mental QoL, which provides interesting information about the impact of physical activity in a diseased population [21]. As previously proven, these results are plausible with published data and might enhance the personalization of lifestyle counselling, using a QoL based evaluation of patients in the consultation room and trying to implement healthy habits in an individualized order, according to QoL needs [40].

The interaction between disease and unhealthy habits in the prediction of Physical and Mental Health suggests that although general advice is necessary in the prevention of health deterioration, lifestyle counselling might have an even more important role in diseased patients. This is one of the most interesting findings of the present research: the interaction between physical or mental comorbidity and unhealthy habits in QoL summaries. This outcome emphasizes the need to accelerate the introduction of the way of life assessment in the patients’ evaluation and treatment and to consider that QoL might rely on dual habits and diseases. In other words, to understand physical and mental domains concerning QoL, it appears to be necessary to take into account that the impact of unhealthy habits affects patients differently depending on their disease burden. 

This study should be considered as a proof of concept, where some limitations should be considered. The cross-sectional design of the study prevents from assessing causality between the different approaches to QoL. In addition, the relatively small sample might reduce the statistical power of the study where type I or type II errors cannot be totally discarded. The low rate of completion of anthropometric data in the auto-declared part of the survey did not allow the evaluation of BMI influence in the results. Additionally, information about the interaction between QoL and BMI has been previously reported in a mostly healthy population [41]. Nevertheless, the comorbid nature of the analyzed cohort, advice to consider the CCI which includes aspects concerning obesity related manifestations and comorbidities instead of BMI. Meanwhile, the consecutive recruitment of the population, the practical approach to lifestyle and the plausibility of the findings, may at least support a further, prospectively designed research on this field. Indeed, the wide inclusion criteria for the enrolment of the participants and the relatively low sample size are limitations of this research. However, the use of validated methods and the plausibility of the results make this study specifically valuable, given the reduced number of studies assessing the screened interactions concerning lifestyle, QoL and comorbidities. Additionally, the emerging interest for a renewed personalized medicine approach demands the evaluation of precision elements related to diet and physical activity in the HRQoL assessment in the clinical setting. 

Precision patient care requires the adaptation of therapeutic measures not only to the patients’ disease, but to the patients themselves [42,43]. In this context, a more personalized evaluation of diseased individuals should be introduced in the regular consultation room to achieve a deeper knowledge level of the patients and to individualize counselling and treatment [32,44]. The clinical implication of the study is to provide useful and cost-effective proof of the association between a simple lifestyle anamnesis and the QoL of patients, which could improve their medical evaluation and management. Indeed, dietary and physical activity factors appeared as relevant determinants of QoL in a heterogeneous morbid population, which has been scarcely investigated so far, despite the clinical implications in hospital guidelines to improve medical care. The proposal of an easy way to examine lifestyle associated with QoL might provide a remarkable tool in the precision medicine scenario. These findings might expand the capacity of evaluating lifestyle in the general medical consultation approach to the patient with cost-effectiveness. Thus, the present research underlines the influence of individual health counselling in the QoL evaluation and improvement. In fact, the enhancement of the association of unhealthy habits in diseased patients should be taken into account as a warning in daily patient management. In this context, the increasing concern for QoL in the general population could be a crossroad between medical doctors and patients in the fight against disease, emphasizing the role of lifestyle in patient health empowerment.

## 5. Conclusions

The lifestyle assessment according to five simple unhealthy habits such as tobacco use, low consumption of raw nuts, overconsumption of carbonated drinks or ultra-processed pastries and lack of physical activity or sleep time, is associated with a worse QoL in the different subscales of SF-36. The interaction between comorbidity and unhealthy habits is especially clear in diseased patients due to the interplay between illness and lifestyle in the prediction of QoL.

## Figures and Tables

**Table 1:** Population demographic, comorbidity, lifestyle and QoL characteristics (n = 262).

Variables	Mean (SD) or n (%)
Age (years)	55.3 (17.5)
Female sex (%)	138 (52.70)
**Comorbidity**	
Myocardial infarction (%)	13 (5.00)
Heart failure (%)	6 (2.30)
Peripheral artery disease (%)	18 (6.90)
Cerebrovascular disease (%)	19 (7.30)
Diabetes mellitus (%)	23 (8.80)
COPD (%)	20 (7.60)
Mild dementia (%)	6 (2.30)
Renal insufficiency (%)	8 (3.10)
Liver disease (%)	17 (6.50)
Tumoral disease (%)	25 (9.50)
Depression (%)	30 (11.50)
CCI (points)	0.91 (1.39)
**Unhealthy habits**	
Smoker (%)	32 (12.20)
Unhealthy dietary habits (%)	109 (41.60)
No raw nuts consumption (%)	146 (55.70)
Absence of physical activity (%)	89 (34.00)
Sleep time less than 7 h (%)	204 (77.90)
**Unhealthy habits (aggregated)**	
4 or more habits (%)	29 (11.10)
3 habits (%)	70 (26.70)
2 habits (%)	97 (37.00)
0 or 1 habit (%)	66 (25.20)
**SF-36 subscales**	
Physical QoL	79.88 (25.73)
Physical role	79.17 (26.01)
Emotional QoL	86,26 (22.17)
Mental QoL	70.74 (19.71)
Social QoL	79.48 (26.76)
Vitality	57.18 (22.00)
Pain	65.88 (25.54)
General QoL	56.01 (20.04)
**SF-36 summaries**	
Physical component of SF-36	65.31 (22.44)
Mental component of SF-36	71.91 (22.46)

**Table 2:** Univariate association of aggregate habits and QoL SF-36 subscales.

SF-36 Subscales	Unhealthy Habits	Mean (SD)	*p*
**Physical QoL**	≥4 habits	59.31 (32.23)	<0.001
	3 habits	75.50 (23.68)	
	2 habits	82.40 (22.66)	
	≤1 habits	91.00 (22.36)	
**Physical role**	≥4 habits	57.97 (35.27)	<0.001
	3 habits	74.55 (26.14)	
	2 habits	82.88 (23.91)	
	≤1 habits	88.65 (16.68)	
**Emotional QoL**	≥4 habits	76.15 (28.32)	<0.001
	3 habits	89.64 (20.63)	
	2 habits	82.47 (24.29)	
	≤1 habits	93.08 (12.97)	
**Mental QoL**	≥4 habits	60.17 (20.15)	<0.001
	3 habits	72.14 (21.65)	
	2 habits	69.58 (20.10)	
	≤1 habits	75.77 (14.56)	
**Social QoL**	≥4 habits	62.93 (31.07)	0.004
	3 habits	78.39 (29.86)	
	2 habits	79.82 (24.89)	
	≤1 habits	87.88 (19.64)	
**Vitality**	≥4 habits	45.26 (23.48)	<0.001
	3 habits	51.61 (23.30)	
	2 habits	59.83 (20.57)	
	≤1 habits	64.81 (18.41)	
**Pain**	≥4 habits	56.09 (22.33)	0.043
	3 habits	62.83 (29.08)	
	2 habits	67.45 (23.89)	
	≤1 habits	70.86 (24.12)	
**General QoL**	≥4 habits	36.55 (18.71)	<0.001
	3 habits	51.86 (20.22)	
	2 habits	58.65 (18.59)	
	≤1 habits	65.00 (15.69)	
**Physical SF-36**	≥4 habits	45.31 (24.81)	<0.001
	3 habits	58.73 (23.01)	
	2 habits	69.88 (19.53)	
	≤1 habits	74.89 (16.99)	
**Mental SF-36**	≥4 habits	63.51 (26.85)	0.019
	3 habits	74.34 (23.98)	
	2 habits	69.24 (23.02)	
	≤1 habits	77.13 (14.89)	

**Table 3:** Multivariate analysis of SF-36 QoL subscales association to the aggregate of unhealthy habits, adjusted by age, sex, CCI and depression.

SF-36 Subscales	Beta for Unhealthy Habits -Per Habit- (SE)	R^2^ for the Model	*p*
Physical QoL	−0.243 (1.407)	0.366	<0.001
Physical role	−0.295 (1.648)	0.158	<0.001
Emotional QoL	−0.149 (1.435)	0.123	0.016
Mental QoL	−0.142 (1.278)	0.115	0.022
Social QoL	−0.179 (1.720)	0.134	0.004
Vitality	−0.254 (1.431)	0.121	<0.001
Pain	−0.152 (1.654)	0.101	<0.001
General QoL	−0.317 (1.199)	0.253	<0.001
Physical SF-36	−0.313 (1.280)	0.310	<0.001
Mental SF-36	−0.118 (1.472)	0.096	0.060

Physical QoL: *p* < 0.05 for age, sex and CCI; Physical role: *p* < 0.05 for CCI; Emotional QoL: *p* < 0.05 for sex and depression; Mental QoL: *p* < 0.05 for depression; Social QoL: *p* < 0.05 for CCI and depression; Vitality: *p* < 0.05 for sex, *p* = 0.06 for CCI; Pain: *p* < 0.05 for sex and depression; General QoL: *p* < 0.05 for CCI and depression; Physical SF-36: *p* < 0.05 for age, sex and CCI; Mental SF-36: *p* < 0.05 for depression.

## Data Availability

The datasets generated for this study can be requested to the corresponding author and would be delivered at reasonable request.
